# Analysis of the aging-induced changes in the motor ability structure using large population fitness test results

**DOI:** 10.18632/aging.202461

**Published:** 2021-01-11

**Authors:** Prabhat Pathak, Siddhartha Bikram Panday, Bong-Keun Jung, Jooeun Ahn

**Affiliations:** 1 Department of Physical Education, Seoul National University, Republic of Korea; 2 Department of Sports and Leisure Studies, Keimyung University, Republic of Korea; 3 Department of Mechanical Engineering, Seoul National University, Republic of Korea; 4 Institute of Sport Science, Seoul National University, Republic of Korea

**Keywords:** motor ability structure, principal component analysis, fitness test results, muscular strength, aerobic endurance

## Abstract

Owing to confounding factors influencing the effect of aging, systematic analyses of age-related changes in motor ability are mostly limited to the use of animals whose diets and genetics can be controlled or the use of datasets of athletes who share similar lifestyles. However, we lack systematic methods for analyzing the effect of aging on the motor ability structure of the general public. We propose that principal component analysis (PCA) on fitness test results of a large sample may provide information on the aging-induced change in the motor ability structure of the general public. We complied the fitness test records of 7402 Koreans between the ages of 20 and 64, and performed PCA on the records of gripping, 50m dash, sit-ups, and shuttle runs, which indicate strength, speed, muscular endurance, and aerobic endurance, respectively. Our analysis shows the structural changes in motor ability around the age of 40 and 60 in Korea. We expect that the proposed approach can be applied to similar datasets from other countries or local communities to quantify any age-induced change in motor ability structure in each specific group.

## INTRODUCTION

Aging is an unavoidable phenomenon, and the resulting gradual decline in motor performance is observed even in the healthy elderly [1] and highly trained master athletes [2–4]. Aging is generally accompanied by a loss of muscle mass, a deterioration of contractile properties of muscles, and a decrease in the maximum rate of oxygen consumption [5–8], which inevitably degrades the motor performance like speed, strength, and endurance. Not only each of these athletic abilities but also the relative dominance of the multiple components changes due to aging [9]. Recently, Panday et al. showed that the dominance shifts from strength to endurance around the age of 50 for highly trained master decathletes [4]. To find the change in the relative dominance, they analyzed the 100m, 400m, and 1500m run records of the decathletes. However, any of these performance records can hardly be obtained from aged non-athletes. We need a systematic method for quantifying the aging-induced changes in the motor ability structure of the general public.

In the past few decades, multiple studies used the muscle biopsy technique to find the causal relationship between aging and motor abilities [10, 11]. Milijkovic et al. found that aging results in the reduction of the size and number of type-2 fibers without significant changes in the type-1 fibers [12]. The significant decrease in type-2 fibers suggests that the reduction in the muscle mass due to aging primarily shifts the dominance from strength to endurance [12, 13]. Further, Deschenes reported that the change in the proportion and size of the muscle fibers begins around the age of 50, and dramatically accelerates around 60 [14].

However, a muscle biopsy is invasive and expensive; analyzing the aging-related changes in motor abilities of a sufficiently large sample using this technique is extremely challenging. Alternatively, a few previous studies used the results of physical fitness tests to analyze changes in the fitness level and motor function depending on age [15, 16]. Among numerous fitness tests, grip strength, 50m dash, sit-ups, and progressive aerobic cardiovascular endurance run (PACER) were used as indicators of the muscular strength, speed, muscular endurance, and aerobic endurance, respectively [17]. The age-dependent change in the results of each test can provide us with knowledge of the effect of aging on each corresponding motor ability.

However, the structure of human motor ability is multi-dimensional, and understanding the effect of aging on human motor ability structure requires more than the separate temporal profile of individual motor function. In this study, adopting the statistical method of principal component analysis (PCA) [18], and consulting previous studies that revealed any group-dependent change in dominant factors using PCA [4, 19–21], we propose a systematic and affordable method for quantifying the aging-induced changes in the motor ability structure of the general public. Applying the proposed method to the data from the National Physical Fitness Test in South Korea, we extracted the dominant principal components (PCs) of the multi-dimensional motor ability structure. Based on the data of 7402 non-athletes whose ages range from 20 to 64, our analysis demonstrates that aging-induced changes in the human motor ability structure occur around the age of 40 and 60 in the case of South Korea.

## RESULTS

We categorized the 7402 South Koreans into the following nine age groups: 20~24, 25~29, 30~34, 35~39, 40~44, 45~49, 50~54, 55~59, and 60~64. The sample size of each age group and their performance in four fitness tests are summarized in Table 1.

**Table 1:** Sample size and performance in four physical fitness tests of the nine age groups.

**Age group**	**20~24**	**25~29**	**30~34**	**35~39**	**40~44**	**45~49**	**50~54**	**55~59**	**60~64**
**Sample size**	1065	768	904	957	833	909	891	668	407
**Grip strength** **(kg)**	34.80 ± 11.42	34.59 ± 11.32	34.68 ± 10.58	35.48 ± 10.69	34.14 ± 9.44	34.08 ± 10.27	32.79 ± 9.13	30.18 ± 8.89	29.22 ± 8.84
**50-meters dash speed** **(m/s)**	5.88 ± 0.97	5.65 ± 0.97	5.42 ± 0.85	5.28 ± 0.84	5.10 ± 0.84	4.95 ± 0.86	4.77 ± 0.85	4.55 ± 0.75	4.08 ± 0.82
**Sit-ups** **(No. of reps)**	39.23 ± 15.59	36.08 ± 14.48	32.99 ± 13.23	32.35 ± 13.30	28.58 ± 11.79	25.49 ± 11.73	23.85 ± 11.84	18.85 ± 10.64	17.34 ± 10.33
**PACER** **(No. of reps)**	43.56 ± 23.89	35.66 ± 17.72	31.82 ± 14.46	29.50 ± 13.31	25.93 ± 10.96	25.24 ± 11.21	22.69 ± 11.02	19.01 ± 8.54	15.39 ± 7.46

The records in each fitness test are shown as mean ± standard deviation.

### Decline in each motor performance

**Figure 1:**
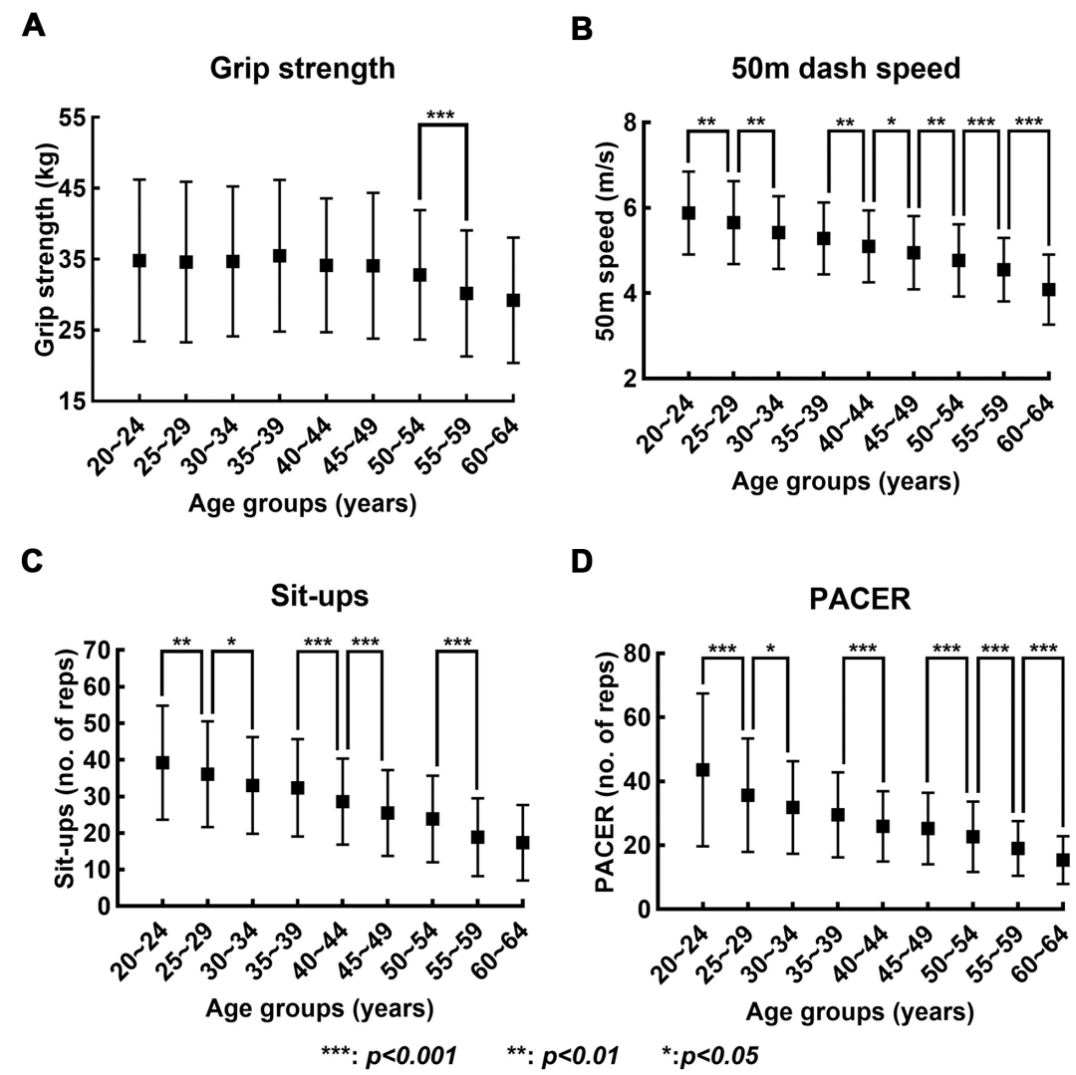
**Changes in records in four fitness tests with increase in age.** (**A**–**D**) show the mean (solid box) and standard deviation (bar) of the records in grip strength, 50-meters dash speed, sit-ups, and PACER, respectively, across the nine age groups.

The average and standard deviation of the records in the four fitness tests of each age group are shown in Figure 1. The results from Kruskal Wallis test revealed that the effect of the age on the performance records was significant for all four fitness tests (grip strength: H(8) = 201.515, p < 0.001; 50m dash: H(8) = 1602.663, p < 0.001; sit-ups: H(8) = 1524.523, p < 0.001; PACER: H(8) = 1508.049, p < 0.001). In the case of grip strength, a statistically significant difference between the pair of neighboring age groups was only found between 50~54 and 55~59 age groups. For 50m dash, the post-hoc comparisons revealed statistically significant differences between all pairs of neighboring age groups except one pair: 30~34 and 35~39 age group. For sit-ups, the post-hoc comparisons revealed statistically significant differences between all pairs of neighboring age groups except three pairs: 30~34 and 35~39; 45~49 and 50~54; and 55~59 and 60~64 age groups. The post-hoc comparisons for PACER revealed statistically significant differences between all pairs of neighboring age groups except two pairs: 30~34 and 35~39; and 40~44 and 45~49 age groups.

**Figure 2:**
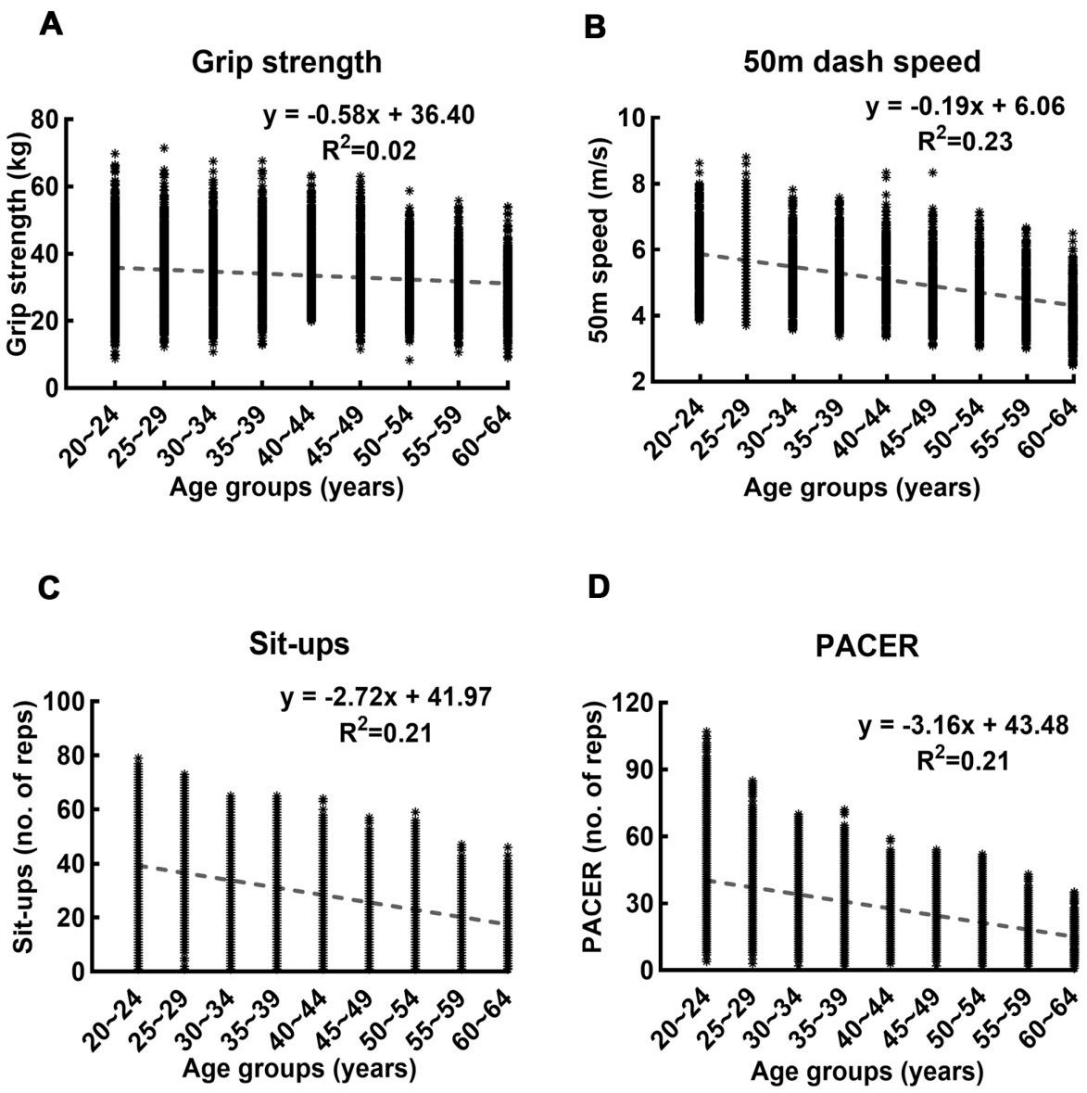
**Linear regression of records in four fitness tests on age.** (**A**–**D**) show the linear regression and the corresponding goodness of fit of the records in grip strength, 50-meters dash speed, sit-ups, and PACER on age, respectively. The R^2^ value of the linear regression of grip strength on age is lower than those of other measures by one order of magnitude.

We additionally performed the linear regression of the records on age for the four fitness tests, and the results are shown in Figure 2. The linear regression equation relating the performance records (*y*) to the age groups (*x*) and the corresponding goodness of fit (*R^2^*) were as follows: grip strength: *y = -0.58x + 36.40, R^2^=0.02*; 50m dash speed: *y = -0.19x + 6.06, R^2^=0.23*; Sit-ups: *y = -2.72x + 41.97, R^2^=0.23*; and PACER: *y = -3.16x + 43.48, R^2^=0.21*.

### Changes in the patterns of factor loadings

**Figure 3:**
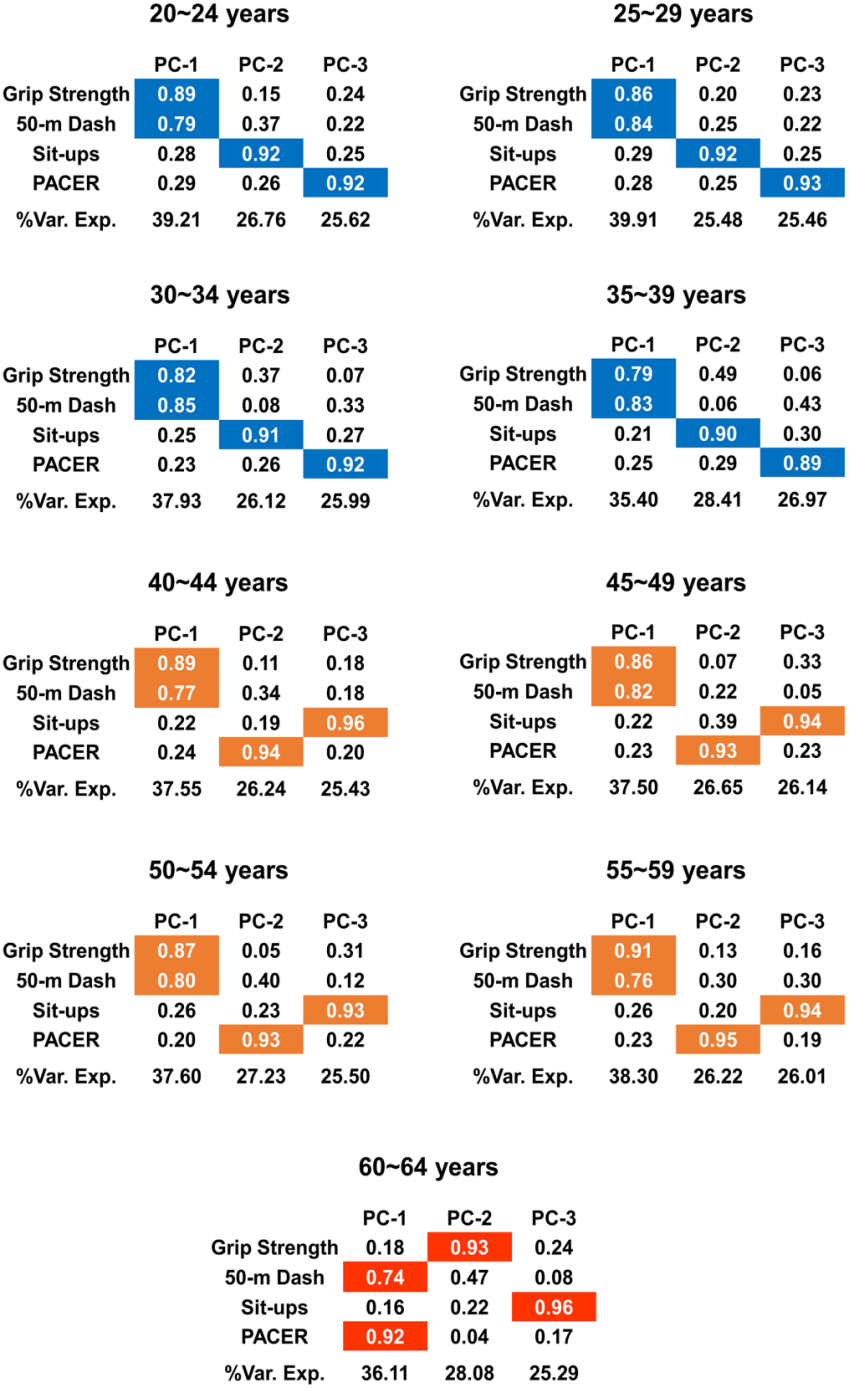
**Changes in the loading patterns with increase in age.** The results from PCA are summarized. Each of the first three PCs and the factor loadings of them are shown for each age group. %Var. Exp denotes the proportion of variance explained by each PC, and the colors are assigned only when the factor loadings are significant, i.e., when the factor loadings are greater than or equal to 0.5. The pattern of the significant factor loadings on the first three PCs changes due to age; the three different colors (blue, orange, and red) indicate three categories classified according to the loading pattern.

Figure 3 shows the factor loadings for the extracted PCs and the percentage of variance explained by each PC. We visualized the loading pattern by highlighting significant factor loadings which are greater than or equal to 0.5. Based on the loading patterns of the PCs, we can classify them into three distinct categories. The loading pattern of the first four age groups, which are 20~24, 25~29, 30~34, and 35~39, shows a high contribution of the grip strength and 50m dash to the formation of the first PC. The sit-ups and PACER have a high contribution to the formation of the second and third PC, respectively. Similar to the first four age groups, the following four age groups, which are 40~44, 45~49, 50~54, and 55~59, still show the dominance of grip strength and 50m dash in the formation of the first PC. However, the dominance of sit-ups and PACER in the formation of the second and third PCs is switched; PACER dominates the second PC, whereas sit-ups dominate the third PC. In the case of the final age group, 60~64, evident changes in the dominance of the four categories of motor performances are observed in the formation of the PCs. The 50m dash and PACER have high contributions to the formation of the first PC, and grip strength and sit-ups dominate the formation of the second and the third PCs, respectively.

We also calculated correlation coefficients by performing the Pearson’s correlation analysis between the four fitness tests for each age group (Supplementary Figure 2). There exists significant correlation between the records of four fitness tests for all age groups (p < 0.001). The correlation coefficient between grip strength and 50m dash was higher than between 50m dash and PACER for age groups between 40 and 59, whereas it was lower for age group 60~64. The results further confirm the change in the loading patterns of the PCs; the significant contribution in the formation of the first PC shifts from grip strength and 50m dash for age groups between 40 and 59 to 50m dash and PACER for age group 60~64.

**Figure 4:**
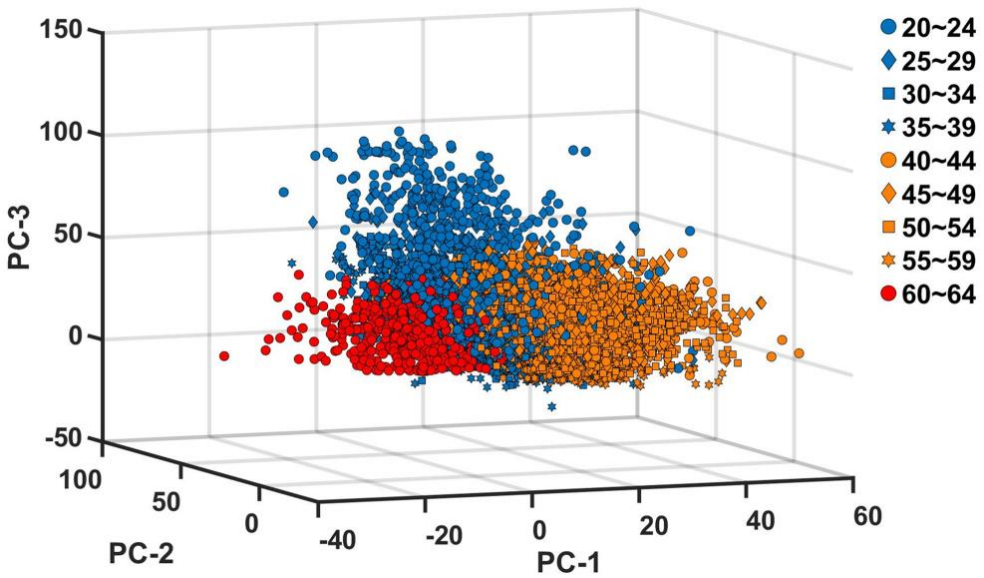
**Projection of principal component scores into the principal component space.** The PC scores are calculated for each of the nine age groups, and projected into the three dimensional space with the basis vectors of PC-1, PC-2 and PC-3 of each age group. The blue, orange and red colors indicate, PC scores of age groups between 20 and 39, PC scores of age groups between 40 and 59, and PC scores of age group 60~64, respectively.

Figure 4 additionally visualizes the changes in the motor ability structure. We calculated the PC scores and projected them into the three dimensional PC space consisting of the axes of PC-1, PC-2 and PC-3 of each age group. The resulting PC score vectors are categorized into three different directions; PCA classifies the nine age groups into three distinct groups both in terms of the direction of PC vectors (Figure 3) and the PC scores in the varied PC vectors (Figure 4).

## DISCUSSION

Quantifying the general tendency of the effect of aging on humans is challenging. Numerous factors, including genetics, social environments, climate, and lifestyle, affect aging, and it is exceptionally challenging to control such factors for human subjects. Hence, systematic experiments on aging have been mostly limited to the use of animal models whose diets and even genetics can be controlled [22–25]. One way to address this challenge is to assess the effect of aging for a relatively homogeneous group; previous studies analyzed the effect of aging on elite athletes considering that this group undergoes a similar level of training and shares a similar diet [2, 4, 26]. However, owing to the homogeneous specificity, the results from such a group can hardly be recognized as the general tendency of human aging. To find any effect of aging on general members of a society despite the various diets and lifestyles of the members, we need a large sample and systematic method. In this study, we suggest one way to achieve this goal. By performing PCA on the fitness test results of 7402 Korean people, we quantified the aging-induced structural change of motor abilities in the case of South Korea.

The variance explained by each PC quantifies the dominance of a specific combination of motor abilities, and the significantly high loading factor on a PC signifies the dominance of the corresponding measure in the formation of the specific PC. Accordingly, our results suggest that muscular strength and speed are dominant up to the age of 59, whereas the dominance shifts from muscular strength to aerobic endurance after 60. Further, from 20 to 39, the second and third PCs are dominated by muscular endurance and aerobic endurance, respectively, whereas the dominance of the second and third PCs are flipped to the aerobic endurance and muscular endurance, respectively from 40 to 59. After 60, the second and third PCs are dominated by muscular strength and muscular endurance, respectively.

Our results reveal that aging may result in a gradual decline in each motor performance (Figures 1, 2) but abrupt changes in the motor ability structure (Figure 3). This contrast between the performance itself and the motor ability structure is consistent with the results from a previous study [4]. The different rates of decline of different motor abilities seem to be partly responsible for this weak correlation between the performance and motor ability structure. Any statistically significant difference between the grip strength records of the neighboring age groups was observed only after the age of 50 (Figure 1), and this is consistent with that the R^2^ value of the linear regression of the grip strength is smaller than those of other motor abilities by one order of magnitude (Figure 2). This substantially lower R^2^ value indicates that a linear model does not explain the decrease in grip strength change with an increase in age; as seen in Figure 1, grip strength remains almost constant up to the age of 50, and then begins to decrease. This result aligns well with the notion that a decline in strength starts around the age of 50, and develops rapidly around 60 [14]. In contrast, the performance records of the rest of the motor abilities are generally inferior to those of the neighboring younger age groups with statistical significance (Figure 1).

One explicit limitation of this study is that analyses were performed only on the data of South Koreans who have a relatively narrow spectrum of genes, and live under the specific climate and social environment in South Korea in recent years. Accordingly, the results of the current study cannot be regarded as the representative tendency of general human aging. However, the methods adopted in this study has important potential. To the best of our knowledge, this is the first study that analyzed the age-dependent changes in each motor ability and relative dominance of each ability based on large population fitness test results of the general public in a specific nation. We expect that similar datasets collected from other countries or local communities may enable the extension of the related research.

In addition to finding the age-induced changes in the motor ability structure and each of the multiple motor abilities, the results can be used to propose a strategy for mitigating age-induced deterioration of motor performance of the general public in each society. For example, muscular strength is a crucial motor ability for the elderly; atrophy results in a decline in muscular strength, which increases the risk of falls [27, 28]. Our study confirms that the dominance of muscular strength substantially declines around the age of 60 in South Korea. By the way, numerous studies have shown that strength training programs can increase muscle mass and proportion of type-2 fibers among the elderly, leading to enhanced muscle strength [29, 30]. Hence, experts in the field of sports science can develop training programs for South Koreans whose age approaches 60. Likewise, a similar analysis method based on PCA can be applied to the dataset from other countries, and the results can be used to design training programs to mitigate any detrimental change in motor ability structure.

## MATERIALS AND METHODS

### Subjects

We extracted the publicly available “National Physical Fitness Test” datasets of South Korea for the years 2011, 2013, and 2015 from https://www.sports.re.kr/front/board/bs/boardList.do?board_seq=48&menu_seq=598 without merging any of the datasets in such a way that individuals might be identified. In addition, this study did not enhance the public data set with identifiable, or potentially identifiable data, which concludes that no approval from the Internal Review Board is needed. We additionally obtained written confirmation that the datasets can be used for this study from the Korean Institute of Sports Science (KISS), who compiled the datasets.

We only included the records in four fitness tests: grip strength, sit-ups, 50m dash, and PACER. The subjects with missing data or zeros in any of the four measures were excluded. The subjects who showed extreme outliers in the records in any of the four measures were also excluded. The outliers were defined as the values that were away from the median by more than three scaled median absolute deviation (MAD) [31]. With this criterion, we classified 417 subjects as outliers. Finally, we selected the records of 7402 subjects for further analysis.

### Description of the fitness tests

The warm-up and physical fitness tests were conducted according to the guidelines provided by the American College of Sports Medicine [17], and the description is written on the official document that is available online (http://sports.re.kr/front/board/bs/boardList.do?menu_seq=48&board_seq=598). The KISS compiled all the records.

### Grip strength

The maximum grip forces of the dominant and non-dominant hands were measured using a standard dynamometer. The participants stood with an upright posture, feet shoulder-width apart, and maintained an angle of 15 degrees between the body and the hand. The participants then held the dynamometer and gripped it as hard as possible. The participants were also asked to avoid bending their elbow and wrist. The test was performed two times each for both hands, and the highest value among the two measurements was recorded as the grip strength. The grip strength was recorded to the nearest 0.1 kg.

### 50m dash

The time record was measured when the participants ran across a course that was 50 meters long and 1.25 meters wide. The start and end lines were marked using chalk or tape. The participants were asked to stand on a starting line, and a research assistant who stood 3~5 meters in front of the course gave the start signal by raising the hand. Another research assistant, who stood next to the finish line, recorded the time gap between the start signal and the moment when the participant reached the finish line. The 50m dash time was recorded to the nearest 0.1 seconds.

### Sit-ups

The maximum number of sit-ups performed by the participants for one minute was measured. The participants were asked to lie on a mat with their feet 30 cm apart and bend their knees at right angles. The participants then placed their hands behind their heads while a research assistant pressed their ankles to prevent the participants from lifting their feet. Another research assistant held a timer and gave the start signal verbally. Once the start signal was given, participants were asked to perform sit-ups by lifting their torso till both of their elbows touched both knees. The sit-ups were not counted if the elbows did not touch the knees.

### Progressive aerobic cardiovascular endurance run (PACER)

The PACER test was performed on a course that is 20 meters long and 1 meter wide. The start and end lines were marked using chalk or tape. The participants ran to the end position and then ran back towards the start position after a beep sound signal was given using a speaker. The time interval between the beep sound signals decreased sequentially so that the average running speed increased by 0.5km/hr after each minute [32]. A triple beep sound signal was given at the end of each minute to alert the participants that the faster speed was going to be needed. If the participants were not able to reach the line before the beep sound, the participants would be given a chance to run back in the opposite direction before the next beep. However, if the participants could not reach the 20-meter mark again before the beep sound, the participants were disqualified. The number of times the participants completed 20 meters run before being disqualified was the record in PACER.

### Data analysis

We averaged the grip strength of the dominant and non-dominant hands. We converted the time record in the 50 m dash into speed because it was reported that the correlation between age and speed is stronger than that between age and time record [4]. Before performing the PCA, we used the Kaiser-Mayer-Olkin (KMO) test for sample adequacy and Bartlett’s test of sphericity to determine the validity of the dataset for the PCA. The results are shown in Supplementary Table 2. By the PCA, we extracted 3 PCs, consulting the results of previous studies [33, 34]. The variance explained by each PC, which was used to select the proper number of PCs, is shown in Supplementary Figure 1. We used the varimax rotation method to identify the PCs, and all of the rotations converged in 4~7 iterations. We performed the Kolgomorov-Smirnov test to assess the normality of the distribution of performance records in the four fitness tests. The Kolgomorov-Smirnov test concluded that none of the data sets was normally distributed (Supplementary Table 1). Therefore, we used the Kruskal Wallis test to assess any statistical difference in the fitness test results across different age groups. The Dunn’s multiple comparison test was performed as a post-hoc analysis. We also performed Pearson’s correlation analysis to assess the strength of linear relationship (correlation coefficient) between records of four fitness tests (Supplementary Figure 2). The level of statistical significance was set at 0.05.

## Supplementary Material

Supplementary Figures

Supplementary Tables
