# Supplementary material for: Analysis of the aging-induced changes in the motor ability structure using large population fitness test results
Source: Aging (Albany NY). 2021 Jan 11;13(1):150–62. doi: 10.18632/aging.202461 (PMC7835041; doi:10.18632/aging.202461)
Supplement: Supplementary Figures [file aging-13-202461-s001.pdf]

SUPPLEMENTARY FIGURES

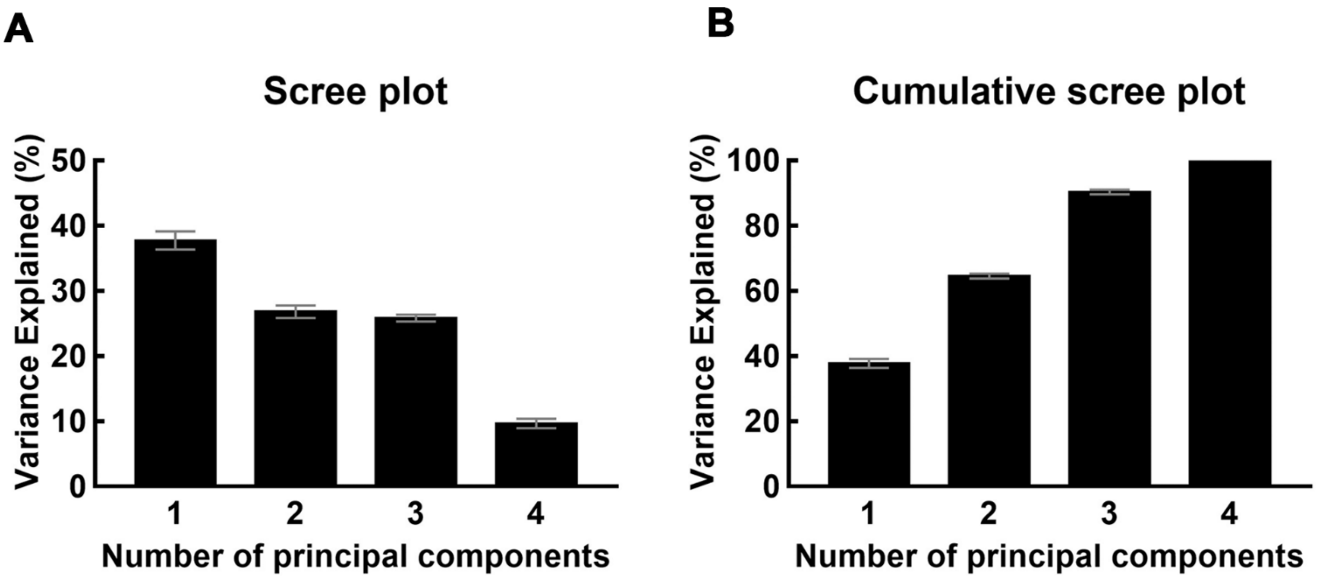

Supplementary Figure 1. The mean and standard deviation of the percentage of variance explained by each principal component (PC) for nine age groups. (**A**, **B**) show the scree plot and cumulative scree plot of the percentage of variance explained by each PC, respectively.

20~24 years

	Grip Strength	50-m Dash	Sit-ups	PACER
Grip Strength	1			
50-m Dash	0.653***	1		
Sit-ups	0.490***	0.561***	1	
PACER	0.507***	0.534***	0.544***	1

25~29 years

	Grip Strength	50-m Dash	Sit-ups	PACER
Grip Strength	1			
50-m Dash	0.634***	1		
Sit-ups	0.498***	0.511***	1	
PACER	0.500***	0.505***	0.550***	1

30~34 years

	Grip Strength	50-m Dash	Sit-ups	PACER
Grip Strength	1			
50-m Dash	0.587***	1		
Sit-ups	0.484***	0.449***	1	
PACER	0.419***	0.452***	0.518***	1

35~39 years

	Grip Strength	50-m Dash	Sit-ups	PACER
Grip Strength	1			
50-m Dash	0.584***	1		
Sit-ups	0.537***	0.449***	1	
PACER	0.479***	0.530***	0.528***	1

40~44 years

	Grip Strength	50-m Dash	Sit-ups	PACER
Grip Strength	1			
50-m Dash	0.551***	1		
Sit-ups	0.382***	0.407***	1	
PACER	0.413***	0.467***	0.425***	1

45~49 years

	Grip Strength	50-m Dash	Sit-ups	PACER
Grip Strength	1			
50-m Dash	0.580***	1		
Sit-ups	0.449***	0.379***	1	
PACER	0.407***	0.484***	0.433***	1

50~54 years

	Grip Strength	50-m Dash	Sit-ups	PACER
Grip Strength	1			
50-m Dash	0.583***	1		
Sit-ups	0.475***	0.462***	1	
PACER	0.359***	0.475***	0.454***	1

55~59 years

	Grip Strength	50-m Dash	Sit-ups	PACER
Grip Strength	1			
50-m Dash	0.606***	1		
Sit-ups	0.441***	0.509***	1	
PACER	0.388***	0.474***	0.436***	1

60~64 years

	Grip Strength	50-m Dash	Sit-ups	PACER
Grip Strength	1			
50-m Dash	0.458***	1		
Sit-ups	0.429***	0.331***	1	
PACER	0.318***	0.542***	0.289***	1

Supplementary Figure 2. Changes in the correlation coefficients between records of the four fitness tests with increase in age. The correlation coefficients are highlighted using three different colors (blue, orange, and red) based on the three categories of PC loading patterns found with increase in age. The triple asterisk (***) indicates statistically significant correlation between records of fitness tests ($p < 0.001$).
