# Supplementary material for: Analysis of the aging-induced changes in the motor ability structure using large population fitness test results
Source: Aging (Albany NY). 2021 Jan 11;13(1):150–62. doi: 10.18632/aging.202461 (PMC7835041; doi:10.18632/aging.202461)
Supplement: Supplementary Tables [file aging-13-202461-s002.pdf]

SUPPLEMENTARY TABLES

Supplementary Table 1. Results of the Kolmogorov-Smirnov normality test.

Age groups	Kolmogorov-Smirnov normality test			
	**Grip strength**	**50m dash speed**	**Sit-ups**	**PACER**
**20~24**	D (1065) = 0.070, $p < 0.001$	D (1065) = 0.053, $p < 0.001$	D (1065) = 0.034, $p = 0.005$	D (1065) = 0.109, $p < 0.001$
**25~29**	D (768) = 0.083, $p < 0.001$	D (768) = 0.073, $p < 0.001$	D (768) = 0.039, $p = 0.006$	D (768) = 0.112, $p < 0.001$
**30~34**	D (904) = 0.081, $p < 0.001$	D (904) = 0.066, $p < 0.001$	D (904) = 0.034, $p = 0.017$	D (904) = 0.091, $p < 0.001$
**35~39**	D (957) = 0.082, $p < 0.001$	D (957) = 0.053, $p < 0.001$	D (957) = 0.039, $p = 0.001$	D (957) = 0.097, $p < 0.001$
**40~44**	D (833) = 0.082, $p < 0.001$	D (833) = 0.034, $p = 0.021$	D (833) = 0.045, $p < 0.001$	D (833) = 0.081, $p < 0.001$
**45~49**	D (909) = 0.084, $p < 0.001$	D (909) = 0.046, $p < 0.001$	D (909) = 0.052, $p < 0.001$	D (909) = 0.120, $p < 0.001$
**50~54**	D (891) = 0.068, $p < 0.001$	D (891) = 0.041, $p = 0.001$	D (891) = 0.048, $p < 0.001$	D (891) = 0.106, $p < 0.001$
**55~59**	D (668) = 0.091, $p < 0.001$	D (668) = 0.062, $p < 0.001$	D (668) = 0.065, $p < 0.001$	D (668) = 0.124, $p < 0.001$
**60~64**	D (407) = 0.071, $p < 0.001$	D (407) = 0.062, $p = 0.001$	D (407) = 0.093, $p < 0.001$	D (407) = 0.087, $p < 0.001$

Supplementary Table 2. Results of the kaiser-mayer-olkin (KMO) test of sample adequacy and bartley's test of sphericity.

Age groups	Kaiser-mayer-olkin (KMO) test	Bartley's test of sphericity
**20~24**	KMO = 0.789	$\chi^2(6) = 1569.079$, $p < 0.001$
**25~29**	KMO = 0.785	$\chi^2(6) = 1059.622$, $p < 0.001$
**30~34**	KMO = 0.760	$\chi^2(6) = 1039.701$, $p < 0.001$
**35~39**	KMO = 0.774	$\chi^2(6) = 1239.683$, $p < 0.001$
**40~44**	KMO = 0.759	$\chi^2(6) = 785.269$, $p < 0.001$
**45~49**	KMO = 0.740	$\chi^2(6) = 939.941$, $p < 0.001$
**50~54**	KMO = 0.749	$\chi^2(6) = 968.833$, $p < 0.001$
**55~59**	KMO = 0.765	$\chi^2(6) = 749.943$, $p < 0.001$
**60~64**	KMO = 0.696	$\chi^2(6) = 337.933$, $p < 0.001$
